# Parametric Analysis and Optimization of Radially Layered Cylindrical Piezoceramic/Epoxy Composite Transducers

**DOI:** 10.3390/mi9110585

**Published:** 2018-11-09

**Authors:** Jianjun Wang, Lei Qin, Weijie Li, Weibin Song

**Affiliations:** 1 Department of Applied Mechanics, University of Science and Technology Beijing, Beijing 100083, China; 2 Beijing Key Laboratory for Sensors, Beijing Information Science & Technology University, Beijing 100101, China; qinlei@bistu.edu.cn; 3 School of Civil and Environmental Engineering, Harbin Institute of Technology, Shenzhen 518055, China; wli27@uh.edu; 4 Department of Mechanics, Beijing Jiaotong University, Beijing 100044, China; 10115246@bjtu.edu.cn

**Keywords:** cylindrical composite, piezoceramic/epoxy composite, electromechanical characteristics, transducer

## Abstract

Radially layered cylindrical piezoceramic/epoxy composite transducers have been designed by integrating the excellent performance of piezoelectric/polymer composites and the radial radiation ability of cylindrical configurations, which are promising in developing novel ultrasonic and underwater sound techniques. Our previous study has explored the effects of the external resistance on the electromechanical characteristics of the transducer, and obtained some valuable findings. To clearly understand the electromechanical characteristics of the transducer and to guide the device design, in this paper, parametric analysis was performed to reveal the effects of multiple key factors on the electromechanical characteristics. These factors include material parameters of epoxy layers, piezoceramic material types, and locations of piezoceramic rings. In order to better analyze the influence of these factors, a modified theoretical model, in which every layer has different geometric and material parameters, was developed based on the model given in the previous work. Furthermore, the reliability of the model was validated by the ANSYS simulation results and the experimental results. The present investigation provides some helpful guidelines to design and optimize the radially layered cylindrical piezoceramic/epoxy composite transducers.

## 1. Introduction

Piezoelectric ultrasonic and underwater sound transducers, by virtue of their excellent electromechanical properties, have been widely applied in medical imaging [1,2,3], non-destructive testing [4,5], underwater communications [6,7], and so on. In recent years, researchers have been trying to improve their design in hope of obtaining more excellent abilities to perform related tasks. Many attempts have been made and this research topic has received more and more attention.

One effective attempt is to add a flexible polymer phase when fabricating the piezoelectric/polymer composites. These composites can overcome the shortcomings of single-phase piezoelectric materials, such as brittleness, high acoustic impedance, and at the same time possess the advantages of both the piezoceramics and the polymers, including low acoustic impedance, high electromechanical coupling coefficient, low mechanical loss and large dielectric constant [8,9]. Through designing and optimizing various connectivity patterns, such as 1-3 type [10,11,12,13,14,15,16,17,18], 2-2 type [3,19,20,21], these transducers can be used in high-frequency, high-power, and high-temperature applications [9]. In addition, an addition of modified epoxy, i.e., aluminum load epoxy, can also enhance their dielectric, piezoelectric and acoustic dampening [22,23,24], which is very useful for structural health monitoring, energy harvesting, and acoustic liners [22].

Another approach is to adopt new structural forms to realize multiple functionalities. The earlier transducers mainly adopted the approximated one-dimensional structures, which can excite one-dimensional longitudinal waves. The representative devices are the longitudinally sandwiched transducers [25,26,27,28,29] that are composed of axially polarized piezoceramic rings and end metal masses. Recently, two dimensional structures, i.e., cylindrical configurations, were proposed to increase the wave coverage area and output power. These structures include tubes, shells, disks and rings [30,31,32,33,34,35,36,37,38,39,40,41,42,43,44,45,46,47]. They can realize the radial wave radiations by utilizing their radial vibrations, and can be used as the omni-directional emitter and receiver in underwater sound and ultrasonic applications [48].

The above two methods can improve the performance of piezoelectric transducers to meet different requirements. However, both of these methods have certain limitations. In addition, in some special applications, such as endoscopic ultrasound (EUS), it is required that the transducer is able to realize 360° imaging [49]. To obtain a comprehensive performance, some efforts have been made to focus on combining the advantages of piezoelectric/polymer composites and those of cylindrical configurations. One attempt was adopting the 1-3 type piezocomposite to design the cylindrical EUS transducers, which can acquire high-resolution EUS imaging [50]. Another attempt was using the 2-2 type piezocomposite to develop the cylindrical underwater acoustic transducers, which can achieve high-frequency wideband ability [51]. In the latest work, a new type of radially layered cylindrical piezoceramic/epoxy composite transducer was developed by integrating two concentric axially polarized piezoceramic rings into cylindrical epoxy matrixes, which is expected to be utilized in developing novel ultrasonic and underwater sound techniques [52]. This preliminary study mainly focused on studying the effects of the external resistance on the electromechanical characteristics of the transducer, which lacked of clear understanding on the influences of other key factors, including material parameters of epoxy layers, piezoceramic material types, and locations of piezoceramic rings. To clearly understand the electromechanical characteristics of this type of transducer and to guide the device design, in this paper, parametric analysis was performed to reveal the effects of these multiple key factors on the electromechanical characteristics of the transducer.

The remainder of this paper is organized as follows. Section 2 exhibits a schematic representation of the radially layered cylindrical piezoceramic/epoxy composite transducer and gives its modified theoretical model based on the model developed in previous work. Section 3 validates the theoretical solution by comparing it to solutions from both ANSYS numerical simulation and experimental investigation. Section 4 discusses the effects of material parameters of epoxy layers, piezoceramic material types, and locations of piezoceramic rings on the electromechanical characteristics of the transducer through numerical analysis. Section 5 draws the conclusions of the paper.

## 2. Modeling

### 2.1. Basic Equations

**Figure 1:**
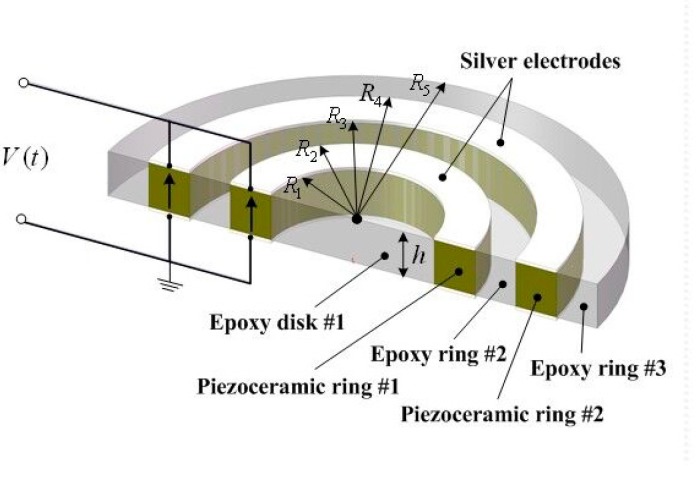
Schematic representation of the radially layered cylindrical piezoceramic/epoxy composite transducer.

Figure 1 exhibits the schematic representation of the radially layered cylindrical piezoceramic/epoxy composite transducer. It consists of a solid epoxy disk, two epoxy rings, and two axially polarized piezoceramic rings. These components are arranged alternatively in the radial direction. The two piezoceramic rings are connected in parallel electrically, and are denoted as piezoceramic ring #1 and piezoceramic ring #2, respectively. Three epoxy layers are denoted as epoxy disk #1, epoxy ring #2 and epoxy ring #3, respectively. The geometric and material parameters of each layer are different. The radial location of the interface between each layer and the axial height of the transducer are defined as $R_{i}\;(i=1,2,3,4,5)$ and h, respectively.

A harmonic form of voltage is used as the excitation source, which is expressed as:

(1) $$V(t)=V_{0}e^{j\omega t},$$

where $V_{0}$, $j=\sqrt{-1}$, ω=2πf, f and t are the excitation amplitude, the imaginary unit, the circular frequency, the excitation frequency and the time, respectively.

Under the assumption of plane stress, the harmonic radial displacement $u_{rP(i)}$, radial stress $\sigma_{rP(i)}$, electric potential $\phi_{\left(i\right)}$ and electric displacement $D_{z(i)}$ of the *i*-th piezoelectric layer (*i* = 1, 2) are expressed as follows [52,53]:

(2) $$u_{rP(i)}=[A_{P(i)}f_{1}(r,i)+B_{P(i)}f_{2}(r,i)]e^{j\omega t},$$

(3) $$\sigma_{rP(i)}=[A_{P(i)}f_{3}(r,i)+B_{P(i)}f_{4}(r,i)+e_{31(i)}(V_{0}/h)]e^{j\omega t},$$

(4) $$\phi_{\left(i\right)}=z(V_{0}/h)e^{j\omega t},$$

(5) $$D_{z(i)}(z)=[A_{P(i)}e_{31(i)}k_{P(i)}J_{0}(k_{P(i)}r)+B_{P(i)}e_{31(i)}k_{P(i)}Y_{0}(k_{P(i)}r)-\kappa_{33(i)}^{\varepsilon }(V_{0}/h)]e^{j\omega t},$$

In Equations (2)–(5), the functions from *f*_1_(*r,i*) to *f*_4_(*r*,*i*) can be expressed as following [52,53]:

(6) $$f_{1}(r,i)=J_{1}(k_{P(i)}r),$$

(7) $$f_{2}(r,i)=Y_{1}(k_{P(i)}r),$$

(8) $$f_{3}(r,i)=c_{11(i)}^{E}k_{P(i)}J_{0}(k_{P(i)}r)+\left[(c_{12(i)}^{E}-c_{11(i)}^{E}\right)/r]J_{1}(k_{P(i)}r),$$

(9) $$f_{4}(r,i)=c_{11(i)}^{E}k_{P(i)}Y_{0}(k_{P(i)}r)+\left[(c_{12(i)}^{E}-c_{11(i)}^{E}\right)/r]Y_{1}(k_{P(i)}r),$$

where $c_{11(i)}^{E}=s_{11(i)}^{E}/\left(s_{11(i)}^{E}s_{11(i)}^{E}-s_{12(i)}^{E}s_{12(i)}^{E}\right)$, $c_{12(i)}^{E}=-s_{12(i)}^{E}/\left(s_{11(i)}^{E}s_{11(i)}^{E}-s_{12(i)}^{E}s_{12(i)}^{E}\right)$, $e_{31(i)}=d_{31(i)}/\left(s_{11(i)}^{E}+s_{12(i)}^{E}\right)$, $\kappa_{33(i)}^{\varepsilon }=\kappa_{33(i)}^{\sigma }-2d_{31(i)}^{2}/\left(s_{11(i)}^{E}+s_{12(i)}^{E}\right)$; $c_{11(i)}^{E}$, $c_{12(i)}^{E}$, $e_{31(i)}$, and $\kappa_{33(i)}^{\varepsilon }$ are the effective elastic, piezoelectric and dielectric constants of the *i*-th piezoceramic layer, respectively. $k_{P(i)}=\omega /V_{rP(i)}$ and $V_{rP(i)}=\sqrt{c_{11(i)}^{E}/\rho_{P(i)}}$ are the radial wave number and sound speed, respectively; $\rho_{P(i)}$ is the density of the piezoceramic. $J_{0}(k_{P(i)}r)$ is the Bessel function of the first kind, and $Y_{0}(k_{P(i)}r)$ is the Bessel function of the second kind.

Similarly, the harmonic radial displacement $u_{rE(i)}$ and radial stress $\sigma_{rE(i)}$ of the *i*-th elastic layers (*i* = 1, 2, 3) are expressed as follows [52,53,54]:

(10) $$u_{rE(i)}(r)=[A_{E(i)}f_{5}(r,i)+B_{E(i)}f_{6}(r,i)]e^{j\omega t},$$

(11) $$\sigma_{rE(i)}(r)=[A_{E(i)}f_{7}(r,i)+B_{E(i)}f_{8}(r,i)]e^{j\omega t}.$$

In Equations (10) and (11), the functions from *f*_5_(*r*,*i*) to *f*_8_(*r*,*i*) can be expressed as following [52,53,54]:

(12) $$f_{5}(r,i)=J_{1}(k_{E(i)}r),$$

(13) $$f_{6}(r,i)=Y_{1}(k_{E(i)}r),$$

(14) $$f_{7}(r,i)=[\left({\bar{E}}_{\left(i\right)}k_{E(i)}\right)/\left(1-\mu_{\left(i\right)}^{2}\right)]\{J_{0}(k_{E(i)}r)+[\left(\mu_{\left(i\right)}-1\right)/\left(k_{E(i)}r\right)]J_{1}(k_{E(i)}r)\},$$

(15) $$f_{8}(r,i)=[\left({\bar{E}}_{\left(i\right)}k_{E(i)}\right)/\left(1-\mu_{\left(i\right)}^{2}\right)]\{Y_{0}(k_{E(i)}r)+[\left(\mu_{\left(i\right)}-1\right)/\left(k_{E(i)}r\right)]Y_{1}(k_{E(i)}r)\},$$

where $k_{E(i)}=\omega /V_{rE(i)}$, $V_{rE(i)}^{2}={\bar{E}}_{\left(i\right)}/\left[\rho_{E(i)}(1-\mu_{\left(i\right)}^{2}\right)]$; $\rho_{E(i)}$, ${\bar{E}}_{\left(i\right)}$ and $\mu_{\left(i\right)}$ are the density, Young’s modulus and Poisson’s ratio of epoxy, respectively.

### 2.2. Solution

As shown in Figure 1, the boundary and continuity conditions of the cylindrical transducer consist of one innermost displacement boundary condition, one outermost stress boundary condition and eight continuous conditions, which are given as follows:

(16) $$\left\{ \begin{array}{l} {u_{rE(1)}|}_{r=0}=0\\ {\sigma_{rE(3)}|}_{r=R_{5}}=0 \end{array}\right.,$$

(17) $$\left\{ \begin{array}{l} {u_{rP(i)}|}_{r=R_{2i-1}}={u_{rE(i)}|}_{r=R_{2i-1}}\\ {\sigma_{rP(i)}|}_{r=R_{2i-1}}={\sigma_{rE(i)}|}_{r=R_{2i-1}} \end{array}\right.\;(i=1,\;2),$$

(18) $$\left\{ \begin{array}{l} {u_{rP(i)}|}_{r=R_{2i}}={u_{rE(i+1)}|}_{r=R_{2i}}\\ {\sigma_{rP(i)}|}_{r=R_{2i}}={\sigma_{rE(i+1)}|}_{r=R_{2i}} \end{array}\right.(i=1,\;2),$$

Combining Equations (2), (3), (10), (11), and (16)–(18), 10 coefficients can be derived, which are listed in Equations (A1)–(A5) (Appendix A). Further, the total electrical charge $Q_{total}(t)$ and the total current $I_{total}(t)$ can be expressed by the following expressions:

(19) $$Q_{total}(t)=Q_{\left(1\right)}(t)+Q_{\left(2\right)}(t)=\int_{0}^{2\pi }\int_{R_{1}}^{R_{2}}D_{z(1)}rd\theta dr+\int_{0}^{2\pi }\int_{R_{3}}^{R_{4}}D_{z(2)}rd\theta dr=({\tilde{C}}_{1}+{\tilde{C}}_{2})V_{0}e^{j\omega t},$$

(20) $$I_{total}(t)=-dQ_{total}(t)/dt=-j\omega ({\tilde{C}}_{1}+{\tilde{C}}_{2})V_{0}e^{j\omega t},$$

where

(21) $${\tilde{C}}_{1}=2\pi \left\{\left(a_{1}v_{9}+v_{1})[f_{9}(R_{2},1)-f_{9}(R_{1},1)]+(a_{2}v_{9}+v_{2})[f_{10}(R_{2},1)-f_{10}(R_{1},1)\right]\right\}-C_{1},$$

(22) $${\tilde{C}}_{2}=2\pi \left\{\left(a_{5}v_{9}+v_{5})[f_{9}(R_{4},2)-f_{9}(R_{3},2)]+(a_{6}v_{9}+v_{6})[f_{10}(R_{4},2)-f_{10}(R_{3},2)\right]\right\}-C_{2},$$

(23) $$f_{9}(r,i)=e_{31(i)}rJ_{1}(k_{P(i)}r)(i=1,2),$$

(24) $$f_{10}(r,i)=e_{31(i)}rY_{1}(k_{P(i)}r)(i=1,2),$$

In Equations (23) and (24), $C_{1}=\pi (\kappa_{33(1)}^{\varepsilon }/h)(R_{2}^{2}-R_{1}^{2})$ and $C_{2}=\pi (\kappa_{33(2)}^{\varepsilon }/h)(R_{4}^{2}-R_{3}^{2})$ are the clamped electric capacitances of piezoceramic rings #1 and #2 in radial vibration, respectively. ${\tilde{C}}_{1}$ and ${\tilde{C}}_{2}$ are the effective electric capacitances of piezoceramic rings #1 and #2 in radial vibration, respectively. Then, the electrical impedance of the transducer Z can be given as:

(25) $$Z=V(t)/I_{total}(t)=-1/\left[j\omega ({\tilde{C}}_{1}+{\tilde{C}}_{2}\right)],$$

Subsequently, by letting |Z|=0 and |Z|=∞, we can obtain the resonance frequency $f_{r}$ and anti-resonance frequency $f_{a}$, respectively. Based on these frequencies, the electromechanical coupling factor of the transducer is obtained as [55]:

(26) $$k_{d}^{2}=\left(f_{a}^{2}-f_{r}^{2}\right)/f_{a}^{2},$$

## 3. Validation

In this section, an ANSYS numerical simulation and an experimental study were conducted to validate the reliability of the theoretical solution. The geometric dimensions of the transducer are given as: *R*_1_ = 10 mm, *R*_2_ = 15 mm, *R*_3_ = 20 mm, *R*_4_ = 25 mm, *R*_5_ = 30 mm, and *h* = 5.63 mm. Materials of the piezoceramic layers were selected as piezoceramic material type (PZT-5H), of which material parameters are listed in Table 1. Three different epoxy materials were chosen for epoxy layers, which have the same Poisson’s ratio, approximately equal density and a certain difference in their Young’s modulus. The material parameters of these three different epoxy materials can be found in Table 2, which are numbered as ①, ②, and ③, respectively. In the following analysis, these geometric dimensions and material parameters will be adopted, unless otherwise stated.

### 3.1. ANSYS Numerical Simulation

**Figure 2:**
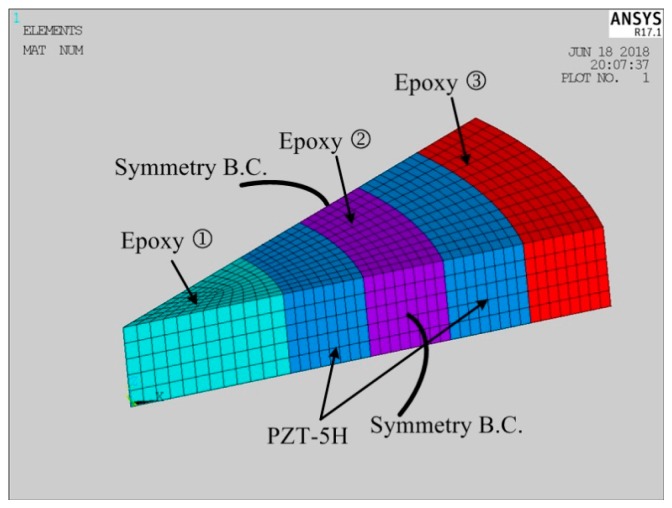
Three-dimensional finite element model of one-twelfth of the transducer.

**Figure 3:**
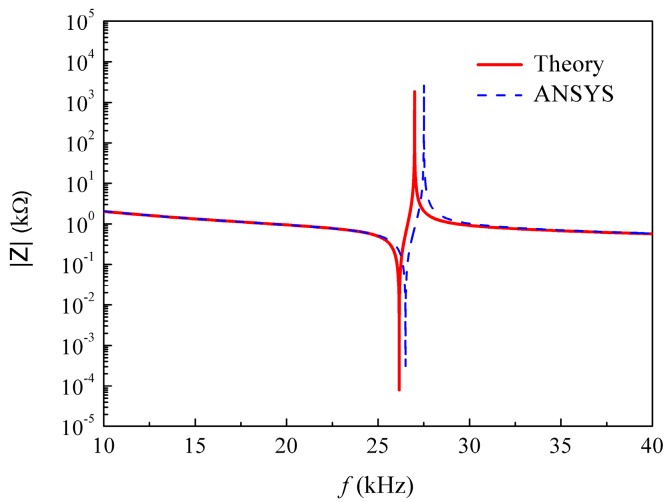
Theoretical and simulated impedance spectra.

In this section, a finite element analysis based on the software ANSYS R17.1 was performed to compare with the theoretical results. A three-dimensional model of one-twelfth of the transducer was created because of the structural symmetry, as shown in Figure 2. In the simulation, the elements, Solid 185 and Solid 5, were used for the epoxy parts and the piezoelectric parts, respectively. The total amounts of elements and nodes were set as 2460 and 3258, respectively, to guarantee the computational precision. All the voltage degrees of freedom (DOFs) of the positive electrodes were coupled together, and the electrical condition *V*_0_ = 1 V was applied. All the voltage DOFs of the negative electrodes were also coupled together, and the electrical condition *V*_0_ = 0 V was applied. The harmonic analysis type was selected, and the frequency range was from 10 kHz to 40 kHz. The simulated impedance–frequency curve is plotted in Figure 3. In addition, the theoretical impedance–frequency relation is also plotted in Figure 3. It can be found that the results from theoretical analysis and finite element analysis agree reasonably well with each other. Further, the theoretical and simulated first resonance and anti-resonance frequencies are compared in Table 3. The relative errors between the theoretical values and the simulated ones for the first resonance frequency and the first anti-resonance frequency are −1.31% and −1.89%, respectively. The above comparative results validate the reliability of the theoretical solution.

### 3.2. Experimental Validation

**Figure 4:**
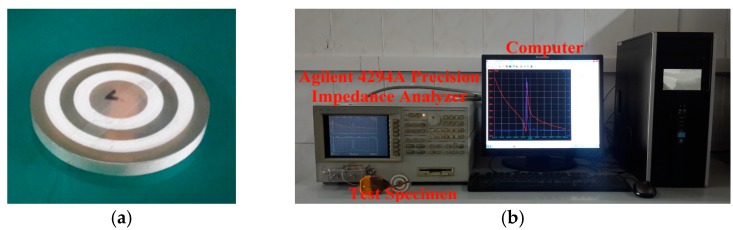
Experimental setup: (**a**) the fabricated specimen; (**b**) the impedance test system.

**Figure 5:**
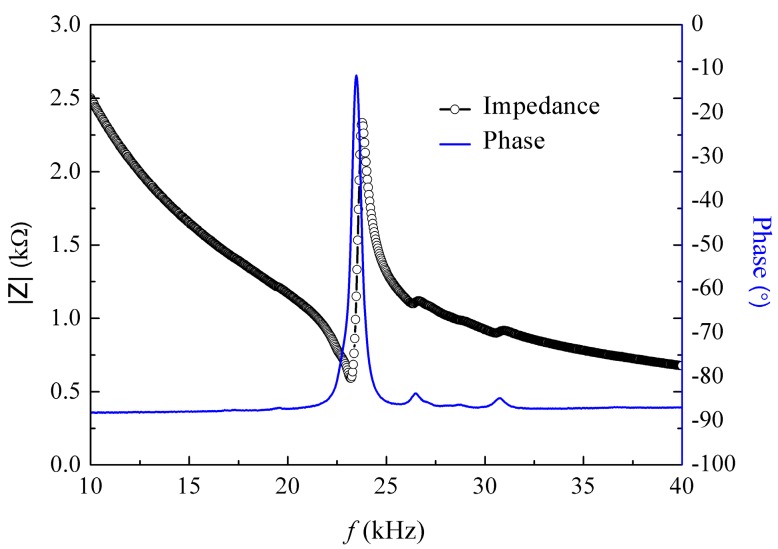
Measured impedance spectrum and its phase.

In this section, a test specimen of the radially layered cylindrical piezoceramic/epoxy composite transducer was fabricated by utilizing the mold-filling technique [52], as shown in Figure 4a. Similar to the previous work [52], the specimen fabrication mainly includes 8 steps: (1) mold and piezoceramic rings preparation, (2) epoxy preparation, (3) pouring epoxy into mould, (4) curing, (5) demolding, (6) polishing, (7) silvering and (8) final specimen. A difference is that in the step (3), three different epoxy materials were poured into the mold in this experiment. Three different epoxy materials, shown in Table 2, were prepared by mixing curing agents of 4,4′-methylenedianiline and bisphenol-A epoxy resin (E-51 with an epoxy value of 0.51 mol/100 g) at a mass ratio of 15/100, 17/100, 19/100, respectively. The curing agents of 4,4′-methylenedianiline was provided by Acros Organics Co. (Geel, Belgium). The E-51 was supplied by Nantong Xingchen Synthetic Material Co., Ltd. (Nantong, China). PZT-5H was selected as the piezoceramic material, shown in Table 1, which was provided by Baoding Hongsheng Acoustics Electron Apparatus Co., Ltd. (Baoding, China). The specimen sizes were same as the given ones. The impedance test system is shown in Figure 4b, which included an Agilent 4294A Precision Impedance Analyzer for measurement and a computer for data acquisition. The electrical parallel connection was realized by using two conductive copper foil tapes. The measured impedance spectra and the phase of the impedance over the frequency range between 10 kHz and 40 kHz are shown in Figure 5. From the spectra, the first resonance and anti-resonance frequencies can be obtained as 23.179 kHz and 23.780 kHz, respectively. These two frequencies are also addressed in Table 3 to compare with the theoretical values. As can be seen, the calculated values are larger than the experimental ones; however, they agree reasonably well with each other. The relative errors between the theoretical values and experimental values for the first resonance frequency and the first anti-resonance frequency are 11.37% and 11.91%, respectively. There are two main factors accounting for the errors. Firstly, the theoretical model was established based on plane stress assumption, which is not the case for the fabricated composite. Secondly, the material parameters provided by the manufacturer were used here and the provided values may not be the exact values of the components used.

## 4. Results and Discussion

In this section, the effects of material parameters of epoxy layers, piezoceramic material types, and locations of piezoceramic rings on the electromechanical characteristics will be analyzed and discussed.

### 4.1. Effect of Material Parameters of Epoxy Layers 

**Figure 6:**
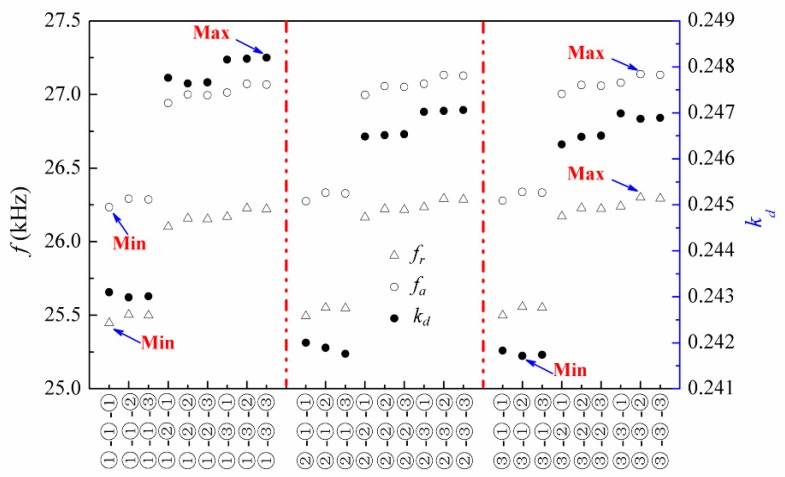
Electromechanical characteristics of the transducers with different material sequences of the epoxy layers.

In the above experiment, the transducer with a sequence of material parameters ①-②-③ for epoxy layers #1, #2 and #3 was fabricated and tested. Here, the sequence ①-②-③ denotes that the material parameters for epoxy layers #1, #2, and #3 are materials ①, ②, and ③, respectively. Keeping the PZT-5H and geometric dimensions of the transducer unchanged, 27 sequences can be formulated according to different material arrangements of these three epoxy layers. Figure 6 plots the electromechanical characteristics for these 27 sequences. These electromechanical characteristics are the first resonance and anti-resonance frequencies and the corresponding electromechanical coupling factors. It can be seen that these 27 different sequences present 27 sets of electromechanical characteristics, which enable the multi-frequency characteristics of the transducer. In addition, transducer with the sequence ③-③-② has the maximum first resonance and anti-resonance frequencies, while the one with the sequence ①-①-① has the minimum frequencies. The transducer with the sequence ①-③-③ has the maximum electromechanical coupling factor, while the one with the sequence ③-①-② has the minimum value. As can be seen from Table 1, since Poisson’s ratios are the same for all these three epoxy layers, the Young’s modulus and density are the contributing factors to the variation in the electromechanical characteristics. The following analysis will discuss their effects on the electromechanical characteristics in order to distinguish the dominant factor.

**Figure 7:**
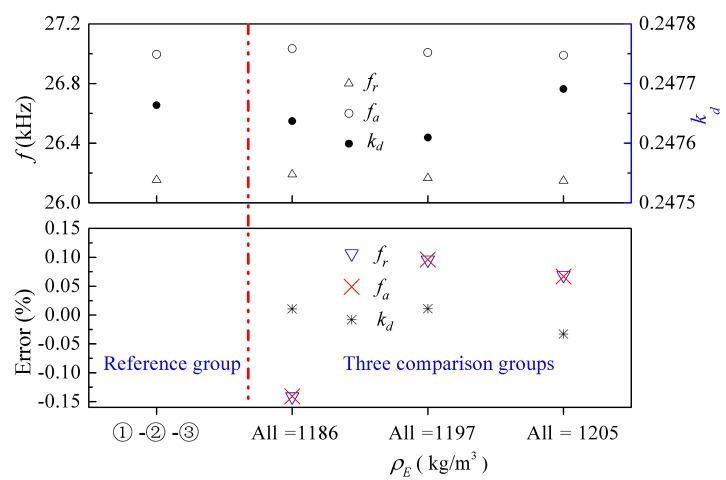
Influence of density of the epoxy layers on the electromechanical characteristics of the transducer. Error = (Reference group − Comparison group)/Reference group.

Keeping other parameters unchanged, Figure 7 presents the effects of variation in density on the electromechanical characteristics. We had one reference group and three comparison groups. Here, the ①-②-③ combination was selected as the reference group, and three special cases with the same density within the group were selected as the comparison groups. From Figure 7, it can be observed that all of the first resonance and anti-resonance frequencies, as well as the corresponding electromechanical coupling factors, are very close to each other. A maximum relative error is −0.14%, which indicates that the effect of density on the electromechanical characteristics is very small and even negligible.

**Figure 8:**
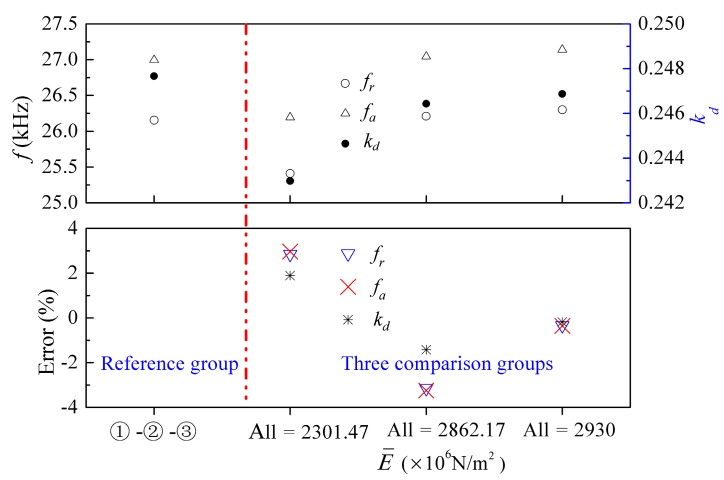
Influence of Young’s modulus of the epoxy layers on the electromechanical characteristics of the transducer. Error = (Reference group − Comparison group)/Reference group.

Similarly, Figure 8 shows the effects of variation in Young’s modulus on the electromechanical characteristics of the transducer. For this case, three special cases with the same Young’s modulus within each group were selected as the comparison groups. The differences between the electromechanical characteristics of these examples can be seen from Figure 8, where the maximum relative error is −3.24%. It is worth noting that this maximum error is 23 times more than that for density, which proves that the Young’s modulus is the dominant factor for the electromechanical characteristics of the transducer. These results can serve as a good reference for designing the transducer.

**Figure 9:**
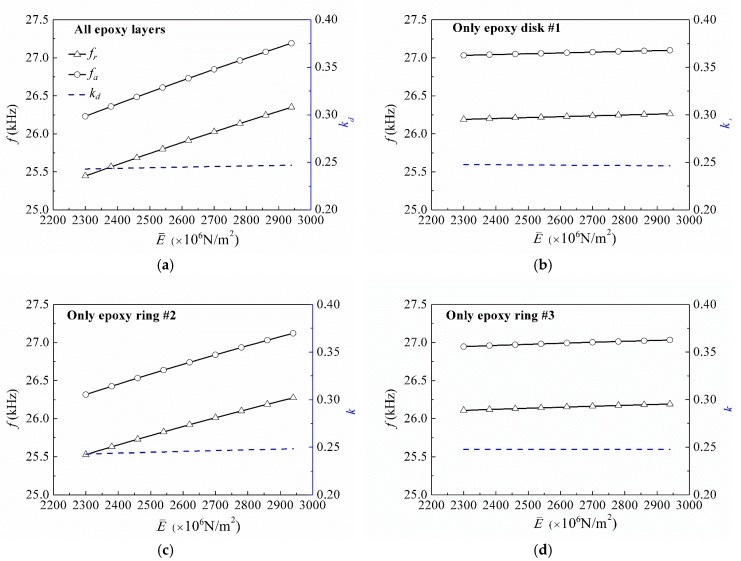
Electromechanical characteristics versus Young’s moduli of the epoxy layers: (**a**) all epoxy layers; (**b**) only epoxy disk #1; (**c**) only epoxy ring #2; (**d**) only epoxy ring #3.

Further, keeping the density of all epoxy layers as 1186 kg/m^3^, Figure 9 plots the variation of the electromechanical characteristics of the transducer when Young’s modulus of epoxy layers changes from 2300 × 10^6^ N/m^2^ to 2940 × 10^6^ N/m^2^. Four cases are presented, i.e., the case of changing all epoxy layers, the case with only epoxy disk #1 changing, the case with only epoxy ring #2 changing, and the case with only epoxy ring #3 changing. It can be seen that the first resonance and anti-resonance frequencies increase with the increase of the Young’s modulus of the epoxy layers. This is because larger Young’s modulus will increase the stiffness of the transducer, which leads to higher resonant frequencies. Furthermore, it can be seen that changing Young’s modulus of epoxy disk #1 and epoxy ring #3 has negligible effects on these two frequencies as compared to the case of changing the Young’s modulus of epoxy ring #2. Therefore, in the transducer design, adjusting the Young’s modulus of epoxy ring #2 can only realize frequency control of the proposed radial layered cylindrical piezoceramic/epoxy composite transducer. From Figure 9, it can also be found that for every case, the variation of Young’s modulus of the epoxy layers has almost no effect on the corresponding electromechanical coupling factors. Here, it should be pointed out that the Poisson’s ratio also greatly influences the electromechanical characteristics of the piezoelectric composites, which has been proved by the previous works [59,60,61]. However, in the present work, the main focus is to design a type of new transducers controlled by Young’s modulus of the epoxy layers. Therefore, three different epoxy materials were chosen for the epoxy layers, which have the same Poisson’s ratio, approximately equal density and a certain difference in their Young’s moduli. The results also demonstrate the feasibility of this design.

### 4.2. Effect of Piezoceramic Material Types

**Figure 10:**
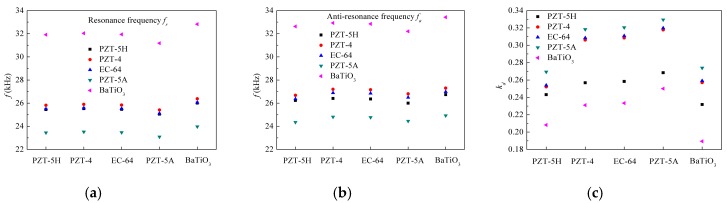
Electromechanical characteristics: (**a**) resonance frequency *f_r_*; (**b**) anti-resonance frequency *f_a_*; (**c**) electromechanical coupling factor *k_d_* versus piezoceramic material types.

Selecting the material parameters of epoxy layers as the sequence ①-①-① and keeping geometric dimensions of the transducer unchanged, Figure 10 gives the effect of combinations of five commonly used piezoceramic materials on the electromechanical characteristics. These piezoceramic materials include PZT-5H, PZT-4, EC-64, PZT-5A and BaTiO_3_, of which material parameters are shown in Table 3. The piezoceramic material types of PZT ring #1 are marked in the abscissa. The piezoceramic material types of PZT ring #2 are listed in the graph. From Figure 10, it can be found that when the PZT ring #2 is chosen as PZT-5A, the transducer has the minimum first resonance and anti-resonance frequencies, but the maximum first electromechanical coupling factor. When the PZT ring #2 is chosen as BaTiO_3_, the transducer has the maximum first resonance and anti-resonance frequencies, but the minimum first electromechanical coupling factor. When the PZT ring #2 are chosen as PZT-5H, PZT-4, EC-64, the transducer has the similar first resonance and anti-resonance frequencies. The reasons are as follows. For a piezoelectric circular ring in radial vibration, when keeping its geometric sizes unchanged, its resonance frequency depends on the radial sound speed $V_{rP}=\sqrt{c_{11}^{E}/\rho_{P}}$ [36]. The radial sound speed reflects its stiffness–mass ratio, of which values are listed in Table 1. A larger $V_{rP}$ for PZT ring #2 means its stiffness is enhanced, which further induces the stiffness increase of the transducer. In addition, its electromechanical coupling effect depends on the plane coupling factor $k_{P(p)}=\sqrt{2d_{31}^{2}/\left[\kappa_{33}^{\sigma }(s_{11}^{E}+s_{12}^{E}\right)}$ [36], as shown in Table 1. A larger $k_{P(p)}$ for PZT ring #2 means its electromechanical coupling effect is better, which further improves the whole coupling effect. The PZT-5A has the minimum $V_{rP}$ and maximum $k_{P(p)}$; therefore, the transducer with PZT-5A ring #2 has the smallest resonance frequency and best electromechanical coupling effect than the other types.

### 4.3. Effect of Locations of Piezoceramic Rings

**Figure 11:**
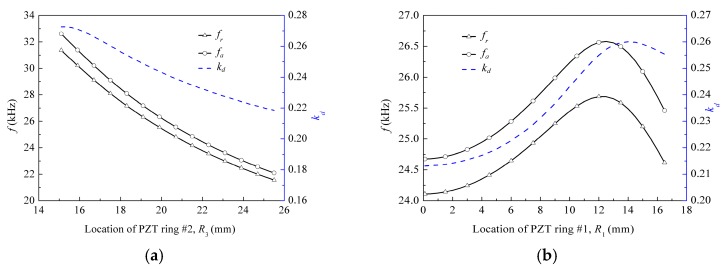
Electromechanical characteristics versus locations of piezoceramic rings: (**a**) location of PZT ring #2; (**b**) location of PZT ring #1.

Selecting the material parameters of epoxy layers as the sequence ①-①-①, piezoceramic material types of two PZT rings as PZT-5H, and keeping the area of one pizeoceramic ring unchanged, Figure 11 shows the relations between the electromechanical characteristics and locations of the other piezoceramic rings. Here, the inner radii *R*_1_ and *R*_3_ of the PZT rings #1 and #2 are used to denote their locations, respectively. The corresponding outer radii *R*_2_ and *R*_4_ of the PZT rings #1 and #2 also need to be changed to maintain the same areas, which are defined as $R_{2}=\sqrt{S_{1}/\pi +R_{1}^{2}}$ and $R_{4}=\sqrt{S_{2}/\pi +R_{3}^{2}}$, respectively. Symbols $S_{1}$ and $S_{2}$ are the areas of the PZT rings #1 and #2, respectively. When fixing the location of PZT ring #1 and varying the location of PZT ring #2, the inner and outer radii *R*_3_ and *R*_4_ of the PZT ring #2, and the areas of the epoxy rings #2 and #3 also vary. When fixing the location of PZT ring #2 and varying the location of PZT ring #1, the inner and outer radii *R*_1_ and *R*_2_ of the PZT ring #1, and the areas of the epoxy disk #1 and ring #2 also vary. From Figure 11, it is indicated that when the location of PZT ring #1 is fixed, the first resonance and anti-resonance frequencies, as well as the first electromechanical coupling factor, decrease with the increase of location of PZT ring #2. When the location of PZT ring #2 is fixed, the first resonance and anti-resonance frequencies, as well as the first electromechanical coupling factor, firstly increase to the maximum values, and then decrease. That is because different locations of one piezoelectric ring relative to the other will change the geometric sizes itself and those of the adjacent epoxy layers. When the material parameters are unchanged, these variations in the geometric sizes will vary their stiffness and mass, which lead to the change in the electromechanical coupling effect of the transducer. In Figure 11b, three maximum values are *f_r_* = 25.685 kHz, *f_a_* = 26.578 kHz, *k_d_* = 0.26, respectively. The corresponding locations are *R*_1_ = 12 mm, 12.5 mm, and 14 mm, respectively. This rule can be used to design the improved transducer that has the maximum first resonance and anti-resonance frequencies as well as the first electromechanical coupling factor.

## 5. Conclusions

A parametric analysis was performed to study the effects of multiple key factors, including material parameters of epoxy layers, piezoceramic material types, and locations of piezoceramic rings, on the electromechanical characteristics of the radially layered cylindrical piezoceramic/epoxy composite transducer. The main results can be concluded as follow.

(1) Based on the presented three different epoxy materials that have the same Poisson’s ratio, approximately equal density and the certain difference in Young’s modulus, the transducer can present 27 sets of electromechanical characteristics by utilizing different material sequences. Furthermore, these electromechanical characteristics are mainly controlled by the Young’s moduli of the epoxy layers, especially for that of the epoxy ring #2. This result demonstrates that only regulating the Young’s modulus of the epoxy layers can realize the design and optimization of the electromechanical characteristics of the transducer.

(2) Among five commonly used piezoceramic materials (PZT-5H, PZT-4, EC-64, PZT-5A and BaTiO_3_), the transducer with the PZT-5A ring #2 has the minimum first resonance and anti-resonance frequencies as well as the maximum first electromechanical coupling factor; the transducer with the BaTiO_3_ ring #2 has the maximum first resonance and anti-resonance frequencies as well as the minimum first electromechanical coupling factor; the transducer with PZT-5H, PZT-4, EC-64 ring #2 has the similar first resonance and anti-resonance frequencies. That is to say, the selections of piezoceramic material types in the ring #2, the piezoceramic materials with the lager radial sound speed and plane electromechanical coupling factor can optimize the electromechanical characteristics of the transducer.

(3) The locations of piezoceramic rings have great effects on the electromechanical characteristics of the transducer, in particular, an appropriate location can be used to optimize the transducer design, making it have the maximum first resonance and anti-resonance frequencies as well as the first electromechanical coupling factor.

## Figures and Tables

**Table 1:** Material parameters of piezoceramic materials [52,56,57,58].

Material Types	Elastic Constant $\left(\times 10^{-12}\;m^{2}/N\right)$		Piezoelectric Constant $\left(\times 10^{-12}\;C/N\right)$	Dielectric Constant	Density ${(\mathrm{kg}/m}^{3})$	Radial Sound Speed (m/s)	Plane Electromechanical Coupling Factor
	$s_{11}^{E}$	$s_{12}^{E}\;$	$d_{31}$	$\kappa_{33}^{\sigma }/\varepsilon_{0}$	$\rho_{P}$	$V_{rP}$	$k_{P(p)}$
PZT-5H	13	−4.29	−186	4500	7450	3404	0.45
PZT-4	12.3	−4.05	−123	1300	7500	3487	0.56
EC-64	12.8	−4.2	−127	1300	7500	3417	0.57
PZT-5A	16.4	−5.74	−171	1700	7750	2994	0.60
BaTiO_3_	8.55	−2.61	−79	1900	5700	4757	0.35

Permittivity of free space: $\varepsilon_{0}=8.85\times 10^{-12}\;F/m$.

**Table 2:** Material parameters of three types of epoxy materials.

Epoxy Types	Young’s Modulus $\left(\times 10^{6}\;{N/m}^{2}\right)$	Poisson’s Ratio	Density ${(\mathrm{kg}/m}^{3})$
	$\bar{E}$	μ	$\rho_{E}$
①	2301.47	0.43	1186
②	2862.17	0.43	1197
③	2930	0.43	1205

**Table 3:** Comparisons between the calculated, simulated and experimental frequencies.

First Resonance and Anti-Resonance Frequencies	Theory (kHz)	ANSYS (kHz)	Error 1 (%)	Experiment (kHz)	Error 2 (%)
*f*r	26.154	26.497	−1.31	23.179	11.37
*f*a	26.995	27.504	−1.89	23.780	11.91

Error 1 = (Theory − ANSYS)/Theory; Error 2 = (Theory − Experiment)/Theory.

## Appendix A

(A1) $$\left\{ \begin{array}{l} A_{E(1)}=v_{9}V_{0}\\ B_{E(1)}=0 \end{array}\right.,$$

(A2) $$\left\{ \begin{array}{l} A_{P(1)}=(a_{1}v_{9}+v_{1})V_{0}\\ B_{P(1)}=(a_{2}v_{9}+v_{2})V_{0} \end{array}\right.,$$

(A3) $$\left\{ \begin{array}{l} A_{E(2)}=(a_{3}v_{9}+v_{3})V_{0}\\ B_{E(2)}=(a_{4}v_{9}+v_{4})V_{0} \end{array}\right.,$$

(A4) $$\left\{ \begin{array}{l} A_{P(2)}=(a_{5}v_{9}+v_{5})V_{0}\\ B_{P(2)}=(a_{6}v_{9}+v_{6})V_{0} \end{array}\right.,$$

(A5) $$\left\{ \begin{array}{l} A_{E(3)}=(a_{7}v_{9}+v_{7})V_{0}\\ B_{E(3)}=(a_{8}v_{9}+v_{8})V_{0} \end{array}\right.,$$

where

(A6) $$\left\{ \begin{array}{l} H_{1}=f_{1}(R_{1},1)f_{4}(R_{1},1)-f_{3}(R_{1},1)f_{2}(R_{1},1)\\ a_{1}=\left[f_{5}(R_{1},1)f_{4}(R_{1},1)-f_{7}(R_{1},1)f_{2}(R_{1},1)\right]/H_{1}\\ v_{1}=\left[(e_{31(1)}/h)f_{2}(R_{1},1)\right]/H_{1}\\ a_{2}=-[f_{5}(R_{1},1)f_{3}(R_{1},1)-f_{7}(R_{1},1)f_{1}(R_{1},1)]/H_{1}\\ v_{2}=-\left[(e_{31(1)}/h)f_{1}(R_{1},1)\right]/H_{1} \end{array}\right.,$$

(A7) $$\left\{ \begin{array}{l} H_{2}=f_{5}(R_{2},2)f_{8}(R_{2},2)-f_{7}(R_{2},2)f_{6}(R_{2},2)\\ a_{3}=\left\{f_{8}(R_{2},2)[a_{1}f_{1}(R_{2},1)+a_{2}f_{2}(R_{2},1)]-f_{6}(R_{2},2)[a_{1}f_{3}(R_{2},1)+a_{2}f_{4}(R_{2},1)]\right\}/H_{2}\\ v_{3}=\left\{f_{8}(R_{2},2)[v_{1}f_{1}(R_{2},1)+v_{2}f_{2}(R_{2},1)]-f_{6}(R_{2},2)[v_{1}f_{3}(R_{2},1)+v_{2}f_{4}(R_{2},1)+e_{31(1)}/h]\right\}/H_{2}\\ a_{4}=-\left\{f_{7}(R_{2},2)[a_{1}f_{1}(R_{2},1)+a_{2}f_{2}(R_{2},1)]-f_{5}(R_{2},2)[a_{1}f_{3}(R_{2},1)+a_{2}f_{4}(R_{2},1)]\right\}/H_{2}\\ v_{4}=-\{f_{7}(R_{2},2)[v_{1}f_{1}(R_{2},1)+v_{2}f_{2}(R_{2},1)]-f_{5}(R_{2},2)[v_{1}f_{3}(R_{2},1)+v_{2}f_{4}(R_{2},1)+e_{31(1)}/h]\}/H_{2} \end{array},\right.$$

(A8) $$\left\{ \begin{array}{l} H_{3}=f_{1}(R_{3},2)f_{4}(R_{3},2)-f_{3}(R_{3},2)f_{2}(R_{3},2)\\ a_{5}=\left\{f_{4}(R_{3},2)[a_{3}f_{5}(R_{3},2)+a_{4}f_{6}(R_{3},2)]-f_{2}(R_{3},2)[a_{3}f_{7}(R_{3},2)+a_{4}f_{8}(R_{3},2)]\right\}/H_{3}\\ v_{5}=\left\{f_{4}(R_{3},2)[v_{3}f_{5}(R_{3},2)+v_{4}f_{6}(R_{3},2)]-f_{2}(R_{3},2)[v_{3}f_{7}(R_{3},2)+v_{4}f_{8}(R_{3},2)-e_{31(2)}/h]\right\}/H_{3}\\ a_{6}=-\{f_{3}(R_{3},2)[a_{3}f_{5}(R_{3},2)+a_{4}f_{6}(R_{3},2)]-f_{1}(R_{3},2)[a_{3}f_{7}(R_{3},2)+a_{4}f_{8}(R_{3},2)]\}/H_{3}\\ v_{6}=-\{f_{3}(R_{3},2)[v_{3}f_{5}(R_{3},2)+v_{4}f_{6}(R_{3},2)]-f_{1}(R_{3},2)[v_{3}f_{7}(R_{3},2)+v_{4}f_{8}(R_{3},2)-e_{31(2)}/h]\}/H_{3} \end{array},\right.$$

(A9) $$\left\{ \begin{array}{l} H_{4}=f_{5}(R_{4},3)f_{8}(R_{4},3)-f_{7}(R_{4},3)f_{6}(R_{4},3)\\ a_{7}=\left\{f_{8}(R_{4},3)[a_{5}f_{1}(R_{4},2)+a_{6}f_{2}(R_{4},2)]-f_{6}(R_{4},3)[a_{5}f_{3}(R_{4},2)+a_{6}f_{4}(R_{4},2)]\right\}/H_{4}\\ v_{7}=\left\{f_{8}(R_{4},3)[v_{5}f_{1}(R_{4},2)+v_{6}f_{2}(R_{4},2)]-f_{6}(R_{4},3)[v_{5}f_{3}(R_{4},2)+v_{6}f_{4}(R_{4},2)-e_{31(2)}/h]\right\}/H_{4}\\ a_{8}=-\{f_{7}(R_{4},3)[a_{5}f_{1}(R_{4},2)+a_{6}f_{2}(R_{4},2)]-f_{5}(R_{4},3)[a_{5}f_{3}(R_{4},2)+a_{6}f_{4}(R_{4},2)]\}/H_{4}\\ v_{8}=-\{f_{7}(R_{4},3)[v_{5}f_{1}(R_{4},2)+v_{6}f_{2}(R_{4},2)]-f_{5}(R_{4},3)[v_{5}f_{3}(R_{4},2)+v_{6}f_{4}(R_{4},2)-e_{31(2)}/h]\}/H_{4} \end{array},\right.$$

(A10) $$v_{9}=-\left[v_{7}f_{7}(R_{5},3)+v_{8}f_{8}(R_{5},3)\right]/\left[a_{7}f_{7}(R_{5},3)+a_{8}f_{8}(R_{5},3)\right].$$
